# Reduction in the rate of postoperative delirium by switching from famotidine to omeprazole in Japanese hepatectomized recipients

**DOI:** 10.1186/s40780-019-0139-1

**Published:** 2019-05-07

**Authors:** Miho Yamasaki, Yusa Fukuda, Aika Tanimoto, Miko Narahara, Yumi Kawaguchi, Hiromi Ushiroda, Saburo Fukuda, Teruo Murakami, Yorinobu Maeda

**Affiliations:** 1 Department of Pharmacy, Chugoku Rosai Hospital, 1-5-1 Hiro-tagaya, Kure, Hiroshima, 737-0193 Japan; 2 Department of Nursing, Chugoku Rosai Hospital, 1-5-1 Hiro-tagaya, Kure, Hiroshima, 737-0193 Japan; 3 Department of Surgery, Chugoku Rosai Hospital, 1-5-1 Hiro-tagaya, Kure, Hiroshima, 737-0193 Japan; 4 Faculty of Pharmaceutical Sciences, Hiroshima International University, 5-1-1 Hiro-koshingai, Kure, Hiroshima, 737-0112 Japan

**Keywords:** Postoperative delirium, Hepatectomy, H_2_ receptor antagonist, Proton pump inhibitor, Japanese version of the NEECHAM confusion scale

## Abstract

**Background:**

Hepatectomy is a highly invasive procedure with a high probability of postoperative delirium. Treatment with antiulcer drugs is indispensable after hepatectomy for anastomotic ulcer management. The clinical pathway for hepatectomy was reviewed and the antiulcer drug used was switched from famotidine, a H_2_-receptor antagonist, to omeprazole, a proton pump inhibitor, owing to the pharmacist’s intervention.

**Methods:**

Hepatectomized recipients over 65 years of age, except in the cases of laparoscopic surgery and intensive care unit entry, were treated with famotidine injections (10 patients) or omeprazole injections (11 patients), and the incidence rates and severity of delirium were compared between the famotidine and omeprazole groups. The delirium after hepatectomy was assessed using the Japanese version of the NEECHAM confusion scale.

**Results:**

The incidence rates of delirium were 90% in the famotidine group and 27.3% in the omeprazole group. Four out of 9 recipients in the famotidine group were injected with haloperidol to treat for delirium, but no recipients needed this treatment in the omeprazole group.

**Conclusions:**

Compared with famotidine, the use of omeprazole was found to be effective in reducing the incidence rate and severity of postoperative delirium in patients undergoing hepatectomy. Pharmacists should actively strive to mitigate the risks of delirium.

## Introduction

Postoperative delirium refers to the mental dysfunction associated with disturbances in consciousness that are of acute onset and triggered by operative stress. It is a prognosis-related factor of quality of life and results in a high strain on the patient’s family and medical staff. It also has negative effects, such as increased medical costs due to prolonged hospital stays; therefore, the implementation of premeasures for dealing with postoperative delirium, including measures for its appropriate assessment and prevention, is necessary. Various factors are known to be risk factors of delirium: drugs, such as those with anticholinergic properties, corticosteroids, meperidine, and sedative hypnotics, and the use of multiple medications (five or more) are also suggested as probable risk factors of postoperative delirium [1, 2].

It was reported that delirium occurs in 10–24% of adult patients who are hospitalized and in 37–46% of patients undergoing an operation. Also, the incidence was even higher, 87%, in the intensive care unit (ICU) [3]. A survey of the incidence of postoperative delirium in elderly patients conducted in the Department of Surgery of Chugoku Rosai Hospital revealed that the incidence of delirium was highest in patients receiving treatment for liver disorders and occurred in 90% of patients undergoing liver treatment [4]. Hepatectomy is a highly invasive surgery compared with many gastroenterological surgeries and involves direct factors such as hepatic encephalopathy and respiratory organ infection-induced hypoxemia. The insertion of multiple drains and waking after the onset of sleep owing to conditions that require complex postoperative management are precipitating factors that also increase the likelihood of postoperative delirium. Treatment with antiulcer drugs, which are indispensable after hepatectomy for anastomotic ulcer management, can also increase the incidence of postoperative delirium.

In an attempt to reduce the incidence of delirium after hepatectomy, a ward pharmacist reviewed the clinical pathway for hepatectomy and suggested that medical doctors switch the antiulcer drug from famotidine, a histamine 2-receptor antagonist (H_2_RA), to omeprazole, a proton pump inhibitor (PPI). Both H_2_RAs, including famotidine, and PPIs, including omeprazole, were reported to induce delirium [5–10]. It was also reported that the incidence of delirium following surgical treatment of esophageal cancer was significantly lower in the PPI (lansoprazole or omeprazole) group than in the H_2_RA (famotidine or ranitidine) group, at 43.3 and 16.7%, respectively [11]. Anticholinergic drugs, such as famotidine, are reported to cause drug-induced delirium [2, 12] and drugs with a strong anticholinergic effect have a strong effect on the brain [13]. In contrast, omeprazole-induced hyponatremic delirium is rarely reported and occurs only in patients treated with omeprazole for at least 3 months [7]. Omeprazole is almost completely metabolized in the liver, mainly by Cytochrome P450 (CYP) 2C19 and partially by CYP3A4. Thus, the hepatic spare ability of each patient was evaluated before surgery, and hepatectomy was only performed on patients who were considered to be able to withstand the surgery. In addition to antiulcer drugs, the opioid fentanyl citrate was also used for analgesia after hepatectomy in all recipients. However, the contribution of fentanyl citrate to postoperative delirium was not considered in the present study, as the risk is reportedly low [14]. With reference to these previously reported data, the switch from famotidine to omeprazole was implemented for patients that underwent hepatectomy in September 2017 in Chugoku Rosai Hospital.

The present study evaluated and compared the incidence rates and severities of postoperative delirium between the famotidine and omeprazole groups in patients receiving hepatectomy who were over 65 years of age. Cases of laparoscopic surgery and ICU entry after hepatectomy were excluded from the analysis of postoperative delirium, as laparoscopic surgery is less invasive than abdominal surgery and ICU admission has an exceptionally high probability of causing delirium. This study was performed to assess the role of pharmacist intervention in decreasing the risk of postoperative delirium in hepatectomy.

## Methods

Hepatectomy, including partial hepatectomy, subsegmentectomy, and segmentectomy, of hepatocellular carcinoma (primary liver cancer) or metastatic liver cancer was performed by abdominal surgery, depending on the number and the size of tumors and the hepatic spare ability in the Department of Surgery of Chugoku Rosai Hospital. The evaluation of the liver damage was performed in accordance with “The general rules for the clinical and pathological study of primary liver cancer, 6th Edition”, edited by Liver Cancer Study Group of Japan, Kanehara & Co., Ltd., Tokyo, Japan (2015). Patients that underwent hepatectomy, except for cases of laparoscopic surgery of hepatectomy (2 patients) and ICU entry after hepatectomy (2 patients), were treated with an antiulcer drug: the famotidine group, comprising 10 patients between 67 and 87 years of age who underwent operations prior to the amended clinical pathway between February and July 2016, and the omeprazole group, comprising 11 patients between 65 and 80 years of age who underwent the operation following the amendment to the clinical pathway change, between September 2017 and March 2018. Patient histories and the presence or absence of delirium onset were compared, and patient characteristics were compared between the famotidine and omeprazole groups with the previously reported risk factors [15]. Before the change was made to the clinical pathway, the dosing schedule consisted of one injection of 20 mg famotidine on the day of operation and two injections of 20 mg famotidine (40 mg/day) daily on the first to third postoperative days. After the change of the clinical pathway, famotidine injections were switched to 20 mg omeprazole injections for 11 patients.

For the treatment of analgesia after hepatectomy, a mixture of ropivacaine hydrochloride (290 mL of Anapeine® injection 2 mg/mL, Aspen Japan, Tokyo, Japan) and fentanyl citrate (10 mL fentanyl injection 0.05 mg/mL, Janssen Pharmaceutical K.K., Tokyo, Japan) was continuously infused through an epidural at a rate of 5 mL/h in 20 patients; for the remaining 1 patient, a mixture of 30 mL saline and 20 mL fentanyl 0.05 mg/mL was injected intravenously at a rate of 1.5 mL/h. Creatine clearance (CLcr) was estimated by using the Cockcroft-Gault equation, serum creatinine concentration (SCr, mg/dL), body weight (kg), and age (years) of each patient.

When recipients experienced postoperative delirium, haloperidol, or Serenace® injection 5 mg (Sumitomo Dainippon Pharma Co., Ltd., Osaka, Japan), was injected. In addition, if necessary, analgesic treatments were further made using flurbiprofen axetil (Ropion® Intravenous 50 mg, Kaken Pharmaceutical Co., Ltd., Tokyo) and/or acetaminophen (acelio® Intravenous Injection 1000 mg, Terumo Co., Tokyo, Japan). The dose of flurbiprofen axetil was 50 mg/time and the dose of acetaminophen was 15 mg/kg for patients with less than 50 kg body weight, and 1, 000 mg for patients with more than 50 kg body weight. These analgesics were administered up to twice daily. The necessity of analgesic treatment was judged by patient’s vital sign such as sweating, tachycardia, elevated blood pressure, and increased respiratory rate, in addition to the complaint from the patient.

Many factors are involved in the onset of delirium. In this study, we evaluated the effects of the following factors:

1. Preoperative factors

The preoperative factors of age, sex, weight, previous medical history (hypertension, diabetes, cerebrovascular disease, respiratory disease, and high alcohol consumption), and the presence or absence of electrolyte abnormalities and hypoalbuminemia were considered. Electrolyte abnormalities were defined as serum Na levels < 130 or > 150 mEq/L and serum K levels < 3.0 or > 6.0 mEq/L. Hypoalbuminemia was defined as serum albumin levels < 3.5 g/dL.

2) Intraoperative and postoperative factors

The intraoperative and postoperative factors of operative time, blood loss, volume of liver resection, and the presence or absence of epidural anesthesia were considered. In addition, the severity of liver damage as a parameter of hepatic reserve, anesthesia time, number of lines and drains, and preoperative use of benzodiazepine soporifics were compared.

The Japanese version of the NEECHAM confusion scale (J-NCS) [16] was used to assess delirium. The recipients were assessed at 10 PM, which was 2 h after treatment with the antiulcer agent, by using the J-NCS from the operation day to the third postoperative day and corresponds to the period during which delirium occurs at a high frequency; scores of 24 or below were defined as representing a state of delirium (Table 1). Patients who underwent laparoscopic surgery of hepatectomy or ICU entry after hepatectomy were excluded from the analysis.

**Table 1:** Each Component and Score Allotment of Japanese version of the NEECHAM Confusion Scale

Subscale 1 Recognition-information processing (14 points)	
▪ Attention: Attention-Alertness-Responsiveness (0–4 points) ▪ Command: Recognition-Interpretation-Action (0–5 points) ▪ Orientation: Orientation-Short-term memory-Thought/Speech content (0–5 points)	
Subscale 2 Behavior (10 points)	
▪ Appearance (0–2 points) ▪ Motor (0–4 points) ▪ Verbal (0–4 points)	
Subscale 3 Physiologic control (6 points)	
▪ Vital function stability (0–2 points) ▪ Oxygen saturation stability (0–2 points) ▪ Urinary continence control (0–2 points)	
Total score (0–30 points)	
▪ 0–19 points: Moderate to severe confusion ▪ 20–24 points: Mild or early development of confusion ▪ 25–26 points: “Not Confused,” but at high risk of confusion ▪ 27–30 points: “Not Confused,” or normal function	

### Ethical considerations

This study was approved by the ethics committee of Chugoku Rosai Hospital (Approval No.: 2018–16). The patients’ data were anonymized to ensure that individuals could not be identified during the survey.

### Statistical analysis

Continuous variables were expressed as the mean ± standard deviation and compared by using the Mann-Whitney *U*-test. The data were compared between the two groups by the Fisher’s exact test or by Steel-Dwass method for nonparametric multiple comparison test. Excel Statcel3P® was used for statistical analyses, and *p*- values of < 0.05 were considered statistically significant.

## Results

Before the clinical pathway was switched, 10 patients who underwent hepatectomy were treated with famotidine (famotidine group) before and after the clinical pathway was switched, 11 patients were treated with omeprazole (omeprazole group). The preoperative risk factors of delirium are shown in Table 2. There were no differences in age, sex, body mass index, degree of liver damage A/B, and underlying diseases, such as hypertension, diabetes, cerebrovascular diseases, and respiratory diseases between the famotidine and omeprazole groups; there were also no differences in terms of alcohol consumption, electrolyte abnormalities, or hypoalbuminemia. The patient intra- and post-operative factors associated with delirium are shown in Table 3. There were no differences in operative time, anesthesia time, blood loss, volume of liver resection, presence or absence of epidural anesthesia, and number of lines and drains between the two groups. These results suggested that there were no differences in patient characteristics between the famotidine and omeprazole groups.

**Table 2:** Comparison of preoperative delirium-related factors between famotidine- and omeprazole-treated groups

	famotidine (*n* = 10)	omeprazole (*n* = 11)	*P* value
Age (year)	77.4 ± 5.3	72.7 ± 6.1	0.073
Gender (male/female)	6/4	10/1	0.126
Body mass Index	23.3 ± 2.80	23.9 ± 2.53	0.597
Hypertension	7	5	0.245
Diabetes	4	6	0.410
Cerebral vascular disease	2	0	0.214
Respiratory disorders	1	4	0.185
Alcohol abuse	2	1	0.462
Abnormal sodium or potassium	3	1	0.256
Hypoalbuminemia	1	0	0.476
Liver damage (A/B)	9/1	10/1	0.738
Benzodiazepine administration^a)^	3	0	0.090
H_2_ receptor antagonist administration^a)^	0	3	0.124

Values are number of patients or mean ± SD

Liver damage was assessed according to “The general rules for the clinical and pathological study of primary liver cancer, 6th Edition”

^a)^These drugs were taken until one day before the hepatectomy surgery

In statistical analyses, Mann-Whitney *U*-test was used for age and body mas index, and Fisher’s exact test was used for other characteristics

**Table 3:** Comparison of intraoperative and postoperative delirium-related factors between famotidine- and omeprazole-treated groups

	famotidine (*n* = 10)	omeprazole (*n* = 11)	*P* value
Operation time (min)	315.4 ± 83.4	328.6 ± 118.1	0.888
Anesthesia time (min)	409.4 ± 78.6	418.1 ± 117.4	0.939
Bleeding volume (mL)	486.0 ± 629.1	386.4 ± 251.7	0.673
Weight of liver resection (g)	102.0 ± 79.0	109.5 ± 95.0	0.728
Patient-controlled epidural analgesia	9	11	0.476
Number of lines (2/3)^a)^	10/0	10/1	0.524
Number of drains (2/3)	10/0	10/1	0.524

Values are number of patients or mean ± SD

^a^ Two lines: 1 line at central vein and 1 line at peripheral vein

Three lines: 1 line at central vein and 2 lines at peripheral vein

Statistical analyses were applied to Mann-Whitney *U*-test and Fisher’s exact test

**Fig. 1:**
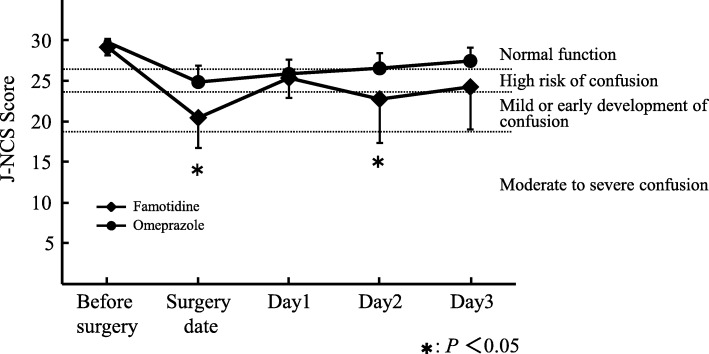
Changes in the J-NCS score. J-NCS score of each recipient was assessed at 10 PM, which corresponds to 2 h after treatment with an antiulcer drug. Mann-Whitney *U*-test was used to analyze the difference in J-NCS scores between famotidine and omeprazole groups

**Fig. 2:**
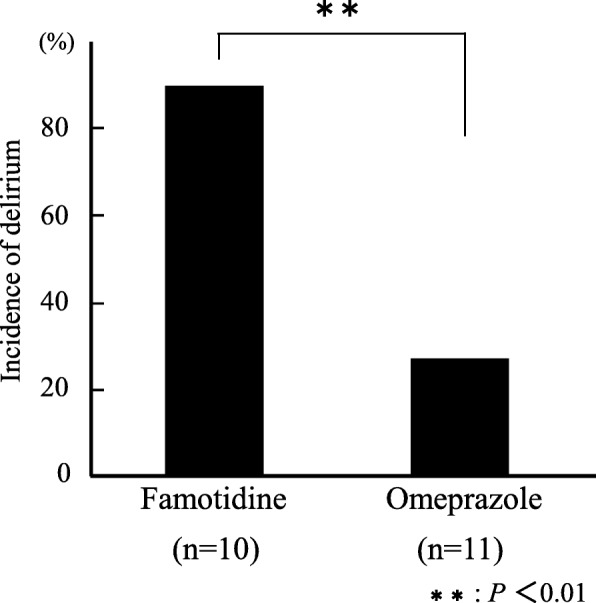
Incidence of postoperative delirium. Fisher’s exact test was used to analyze the difference in incidence of postoperative delirium between famotidine and omeprazole groups

A comparison of the J-NCS scores revealed that preoperatively, there were no differences between the famotidine and omeprazole groups, with scores on the day before operation being 29.3 ± 0.82 and 29.8 ± 0.40 points, respectively. However, on the day of the operation and thereafter, the omeprazole group presented a higher J-NCS score than the famotidine group and presented a significantly higher score on the day of operation and on the second postoperative day (Fig. 1). Nine out of the 10 patients (90%) in the famotidine group were assessed to have delirium, in contrast with three out of the 11 patients (27.3%) in the omeprazole group, which indicated that the incidence of delirium in the omeprazole group was significantly lower than that in the famotidine group (*P* < 0.01) (Fig. 2). Furthermore, five patients in the famotidine group had J-NCS scores of 19 points or below, indicating moderate to severe confusion, and four of these patients required administration of haloperidol for the treatment of postoperative delirium. In contrast, in the omeprazole group, no patients scored 19 points or lower on the J-NCS or were administered antipsychotics. Also, serological examination of each patient indicated that there were no cases to doubt the drug-induced liver injury more than postoperative change in omeprazole group. Significant differences were observed in subscales 1 and 2 of J-NCS scores between the famotidine and omeprazole groups (Table 1), suggesting that the severity of delirium induced in the famotidine-treated group was more serious than that in the omeprazole-treated group with respect to the recognition-information processing and behavior (Table 4). In addition, analgesic treatment was made using flurubiprofen axetil and acetoaminophen in 15 out of total 21 patients. However, this analgesic treatment did not affect the incidence rates of delirium observed in the present study, because the treatment was made in all patients after showing their lowest J-NCS scores. Regarding the length of the stay in hospital, there was no difference between famotidine and omeprazole groups.

**Table 4:** Postoperative J-NCS score in famotidine and omeprazole groups

J-NCS score	0 ~ 19	20 ~ 24		25 ~ 26	
	famotidine (*n* = 5)	famotidine (*n* = 4)	omeprazole (*n* = 3)	famotidine (*n* = 1)	omeprazole (*n* = 8)
subscale 1 (14 points) (Recognition-information processing)	7.4 ± 1.7	11.0 ± 2.0	11.0 ± 1.0	14	13.8 ± 0.46
subscale 2 (10 points) (Behavior)	5.6 ± 3.0	7.8 ± 0.96	7.7 ± 0.58	9	9.6 ± 0.52
subscale 3 (6 points) (Physiologic control)	2.2 ± 0.84	2.8 ± 0.50	3.3 ± 1.2	3	2.3 ± 0.46

J-NCS score was evaluated at 10 PM each day (total 4 days) after hepatectomy, and the lowest score in each recipient was used

The J-NCS score of 24 or less was defined as the condition of delirium onset

Significant difference (*P* < 0.05): In subscale 1, famotidine(0~19) vs omeprazole (25~26), and omeprazole (20~24) vs omeprazole (25~26)

In subscale 2: famotidine(0~19) vs omeprazole (25~26),: famotidine(20~24) vs omeprazole (25~26), and omeprazole (20~24) vs omeprazole (25~26)

In subscale 3: no significant difference each other

Statistical analyses were made by using Steel-Dwass method for nonparametric multiple comparison test

## Discussion

Postoperative delirium is defined as a syndrome in which operative stress triggers the acute onset of a reversible disturbance of consciousness and cognitive impairment; it is characterized by an alternating range of psychiatric symptoms, such as disorientation, sensory illusions, visual hallucinations, delusions, and agitation [17]. Delirium can impose a major strain on patients, their families, and medical care providers alike; therefore, the implementation of measures, including appropriate assessment and prevention, is required. The NEECHAM Confusion Scale (NCS) developed by Neelon et al. [18] is a tool that can be introduced into the daily nursing practice that enables nurses to efficiently screen patients for delirium. It has been shown to have good validity and reliability for the assessment of delirium, and a Japanese version, the J-NCS, was created by Watanuki, et al. [16]. The J-NCS contains nine items in three subscales on information processing and cognitive status (three items), behavior (three items), and physiological control (three items). The risk factors for the development of delirium are categorized into predisposing, precipitating, and direct factors, with delirium understood to occur owing to a combination of several of these factors. Drugs are categorized as a direct factor influencing delirium [19]. In particular, it has become increasingly clear that changes in cholinergic and dopaminergic neurotransmitters are implicated in the onset of delirium [20]. The Clinical Guideline for the Treatment of Delirium, 2nd edition, given by The Japanese Society of General Hospital Psychiatry also lists drugs with anticholinergic effects and γ-aminobutyric acid (GABA) agonists as drugs that can cause delirium. Preventive strategies against delirium involve the identification of risk factors for delirium and their elimination. Avoiding the use of such drugs before operations should, therefore, lower the risk of postoperative delirium. In the present study, three patients in the famotidine group were taking benzodiazepines preoperatively (Table 2). All three patients experienced postoperative delirium after hepatectomy and famotidine treatment; one of these patients was administered haloperidol to treat delirium. In the present study, the relationship between preoperatively-administered benzodiazepine and postoperative delirium was not clear because of the small number of patients. With regard to benzodiazepines, it has been reported that higher doses of preoperatively-administered benzodiazepines are associated with higher risk of delirium [21]. In the omeprazole group, three patients were taking H_2_RA preoperatively; one of these patients scored 24 points or lower on the J-NCS after hepatectomy and omeprazole treatment.

With regard to the postoperative delirium in patients that underwent hepatectomy, a previous study conducted at our hospital revealed that patients who underwent hepatectomy had a high rate of delirium (90%) compared with other gastroenterological surgeries [4]. Thus, the ward pharmacist highlighted the necessity of preventive measures against delirium, reviewed the clinical pathway for hepatectomy, and hypothesized that the administration of famotidine injection to prevent gastric tract bleeding was a risk factor. The usefulness of PPIs, such as omeprazole, compared with H_2_RAs, such as famotidine, for reducing postoperative delirium was already investigated for patients with esophageal cancer by Fujii et al. [11], who reported that the delirium incidence in the H_2_RA and PPI groups was 43.3 and 16.7%, respectively. In good agreement with their report [11], we also observed the greater usefulness of omeprazole than famotidine reducing the incidence rate of postoperative delirium, even in patients undergoing hepatectomy, in which the delirium incidence was 90% in the famotidine group and 27.3% in the omeprazole group. A large difference was observed in the incidence rates of postoperative delirium between postoperative patients with esophageal cancer and hepatectomy. The reason for the larger difference in the incident rates of postoperative delirium between patients with esophageal cancer and patients undergoing hepatectomy, especially in the H_2_RA group (i.e., including famotidine), was not clear. However, the liver disorders could be considered to have a higher probability of causing postoperative delirium compared with other gastroenterological surgeries, as reported previously [4].

Famotidine is mostly excreted into the urine (approximately 80% of the dose) as an intact form and the disposition half-life from plasma is known to be significantly prolonged in patients with mild renal insufficiency (creatine clearance [CLcr] 30–60 mL/min) compared with patients with normal renal function (CLcr > 60 mL/min) [22]. In the famotidine group, 5 out of 10 patients had CLcr of 45–60 mL/min, and the recommended dose of famotidine for such patients with mild renal insufficiency is a half dose of patients with normal renal function. However, in the present study, the serum creatinine concentration (Scr) of four of these patients, (i.e., all except one patient), was within the normal range (0.6–0.7 mg/dL), although the CLcr values estimated by using Cockcroft and Gault equation were below 60 mL/min, possibly due to the patients’ low body weight. In contrast, the remaining patient had a Scr of 1.39 mg/dL, which suggested mild renal insufficiency, and their estimated CLcr was 42.7 mL/min. However, the dose of famotidine for recipients with mild renal insufficiency was not decreased, as it has been reported that famotidine is essentially free of dose-related adverse effects, and that dose adjustment is not required in patients with mild renal insufficiency and in elderly patients, except the case of long-term treatment by Lin et al. [23]. In the present study, the period of famotidine treatment was only 3.5 days (total 7 times dosing). In addition, it is reported that the estimated brain Kp (tissue-to-plasma partition coefficient) values of famotidine in patients with renal disease are comparable with that in subjects with normal hepatic/renal function (Kp = 0.05–0.09), different from the cases of patients with hepatic disease. The Kp values of patients with hepatic disease are reported to be increased by almost three-fold of subjects with normal renal/hepatic function (Kp = 0.14–0.25) [24]. Nine out of the 10 patients in the famotidine group experienced postoperative delirium, irrespective of their CLcr value and age. In contrast, the patient with Scr of 1.39 mg/dL and the lowest CLcr value (42.7 mL/min) in famotidine group did not experience postoperative delirium. About 70% of famotidine given intravenously is excreted into urine as an intact form, and the elimination half-life of famotidine in patients with renal failure is prolonged by about 7- to 10-fold as compared with those in subjects with normal renal function [25]. Lin et al. also reported that a prolonged dosing interval or a decrease in daily dose during long-term therapy may be adapted for the patients with severe renal insufficiency to avoid accumulation and the potential undesirable effects [23]. Although the contribution of renal failure in the induction of postoperative delirium was not observed in a hepatectomized recipient treated with famotidine in the present study, careful dosage adjustment would be necessary when using famotidine in renal failure patients as reported by various articles including package insert for famotidine. After the clinical pathway was changed from famotidine to omeprazole, a survey on the incidence of post-switch delirium was conducted to assess the validity of the change, and the results were compared with those of previous studies. There were no differences in preoperative, intraoperative, and postoperative factors of delirium between the patients in famotidine and omeprazole groups (Tables 2 and 3). Compared with patients in the famotidine group, the proportion of patients with delirium in omeprazole group was significantly lower (90 and 27.3%, respectively; *P* < 0.01). The J-NCS scores of the two groups were approximately equal up to the day before the operation, but postoperative J-NCS scores in omeprazole group were consistently higher than those in the famotidine group, with a significant difference on the day of operation and on the second postoperative day (Fig. 1). In the score of J-NCS on the operation day, the effect of operation itself could be involved. In the present study, the operation time of hepatectomy was about 5 h and anesthesia time was about 7 h (Table 3), and then antiulcer drugs were administered. After about 2 h later, the J-NCS score was evaluated at 10 PM. There were variations in the degree of awakening from anesthesia, or in the depth of anesthesia, among hepatectomized recipients even at 10 PM, which would induce the scattering of the J-NCS scores on the operation day. Five patients in the famotidine group scored 19 points or lower on the J-NCS, which was indicative of moderate to severe confusion (Table 1). Among them, four patients required treatment with haloperidol injection. In contrast, in the omeprazole group, no patients scored 19 points or below on the J-NCS or required treatment with haloperidol injection. This suggested that, collectively, the omeprazole group scored more highly on the J-NCS than the famotidine group and that their delirium was less severe.

Pain is one of risk factors that induce delirium [19]. In this study, a mixture of fentanyl citrate and ropivacaine hydrochloride was administered by constant rate infusion through an epidural to all patients to attempt relief of the postoperative pain. In addition, if the patient is complaining of pain, flurubiprofen axetil and/or acetoaminophen was further administered based on patient’s vital sign. In the famotidine group (total 10 patients), 5 patients received flurubiprofen axetil and 1 patient received acetaminophen and flurubiprofen. In the omeprazole group (total 11 patients), 8 patients received flurubiprofen axetil and 1 patient received acetaminophen. Analgesic treatments were performed after patients showed their lowest J-NCS scores, and therefore did not affect the incidence rates of delirium obtained in the present study. That is, in the case of patients who underwent postoperative delirium, analgesics were administered after the onset of delirium.

Delirium occurs at higher rates in elderly patients, and the risk of delirium increases dramatically in individuals of 65 years or age or more; for each additional year of age, the incidence of delirium is reported to increase by 2% [21]. In the present study, there was no significant difference in the age of patients in the famotidine and omeprazole groups (Table 2). In the famotidine group, 9 out of 10 patients experienced postoperative delirium, regardless of age (67–87 years of age) and renal function (> 45.9 mL/min). In the omeprazole group (65–80 years of age), three out of 11 patients – the two second youngest patients (66 years of age) and the oldest patient in the group (80 years of age) – experienced postoperative delirium. To consider the effect of age of recipients on the onset of postoperative-delirium after hepatectomy, further study is necessary by using data of larger size recipients.

## Conclusions

The use of omeprazole instead of famotidine was found to be effective for the reduction of the incidence rate and severity of postoperative delirium in patients undergoing hepatectomy. There was no difference in the length of the stay in hospital between famotidine and omeprazole groups. Revision of the clinical pathway by the pharmacist and switching drugs that are risk factors of postoperative delirium appear to decrease the incidence rate and severity of delirium. The continued active involvement of pharmacists and strategies to reduce the risk of delirium are needed.

## Abbreviations

CLcr: Creatine clearance
CYP: Cytochrome P450
GABA: γ-aminobutyric acid
H_2_RA: Histamine 2-receptor antagonist
ICU: Intensive care unit
J-NCS: Japanese version of the NEECHAM confusion scale
PPI: Proton pump inhibitor
Scr: Serum creatinine concentration
